# Supplementary material for: Not so cold after all: tumor infiltrating CD8+ T cells in EBV-positive Burkitt lymphoma are quiescent, not exhausted
Source: bioRxiv. 2026 Apr 19:2026.04.15.718702. Preprint. [Version 1] doi: 10.64898/2026.04.15.718702 (PMC13104861; doi:10.64898/2026.04.15.718702)
Supplement: Supplement 3 [file media-3.docx]

**Supplementary Table 3: IHC sample characteristics**

| **Group** | **N** | **Age (median/range)** | **Sex (%Male)** | **Tumor staging (I)** | **Tumor staging (III)** | **Tumor staging (IV)** | **Survival outcome (deceased)** |
| --- | --- | --- | --- | --- | --- | --- | --- |
| EBV^pos^BL | 15 | 9 [3-14] | 80% | 29% | 64% | 7% | 40% |
| HL | 13 | 8 [1-12] | 61% |  |  |  | 33% |
