# Supplementary figures and images for: Not so cold after all: tumor infiltrating CD8+ T cells in EBV-positive Burkitt lymphoma are quiescent, not exhausted
Source: bioRxiv. 2026 Apr 19:2026.04.15.718702. Preprint. [Version 1] doi: 10.64898/2026.04.15.718702 (PMC13104861; doi:10.64898/2026.04.15.718702)

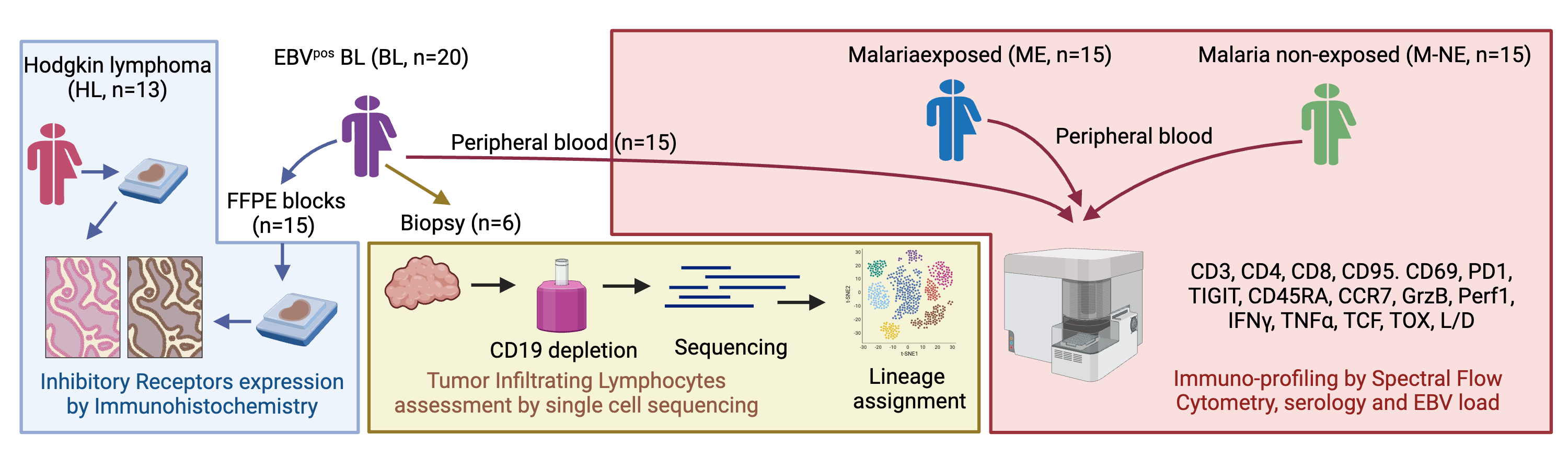

Supplement: Supplement 11 [file media-11.jpg]

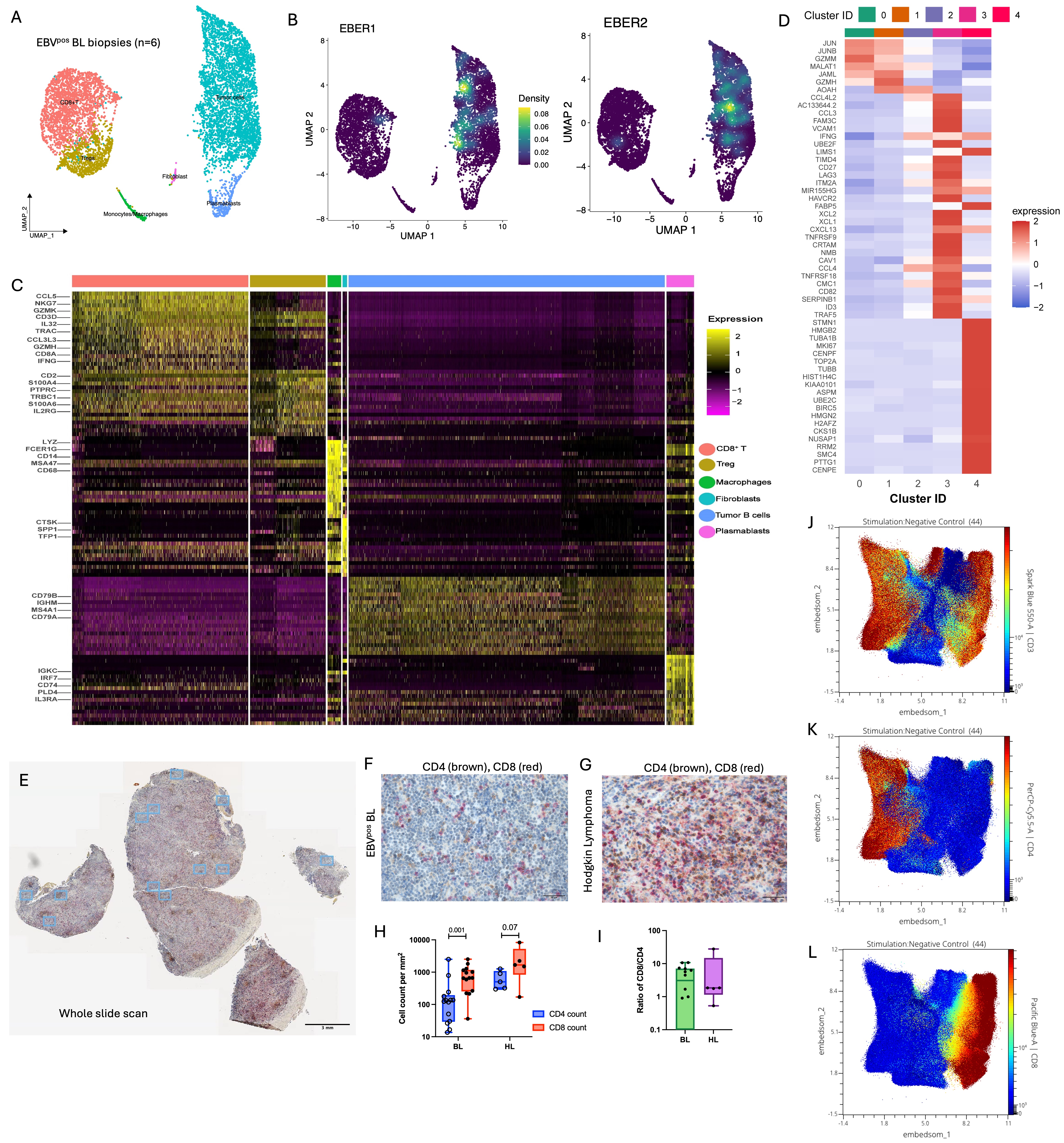

Supplement: Supplement 12 [file media-12.jpg]

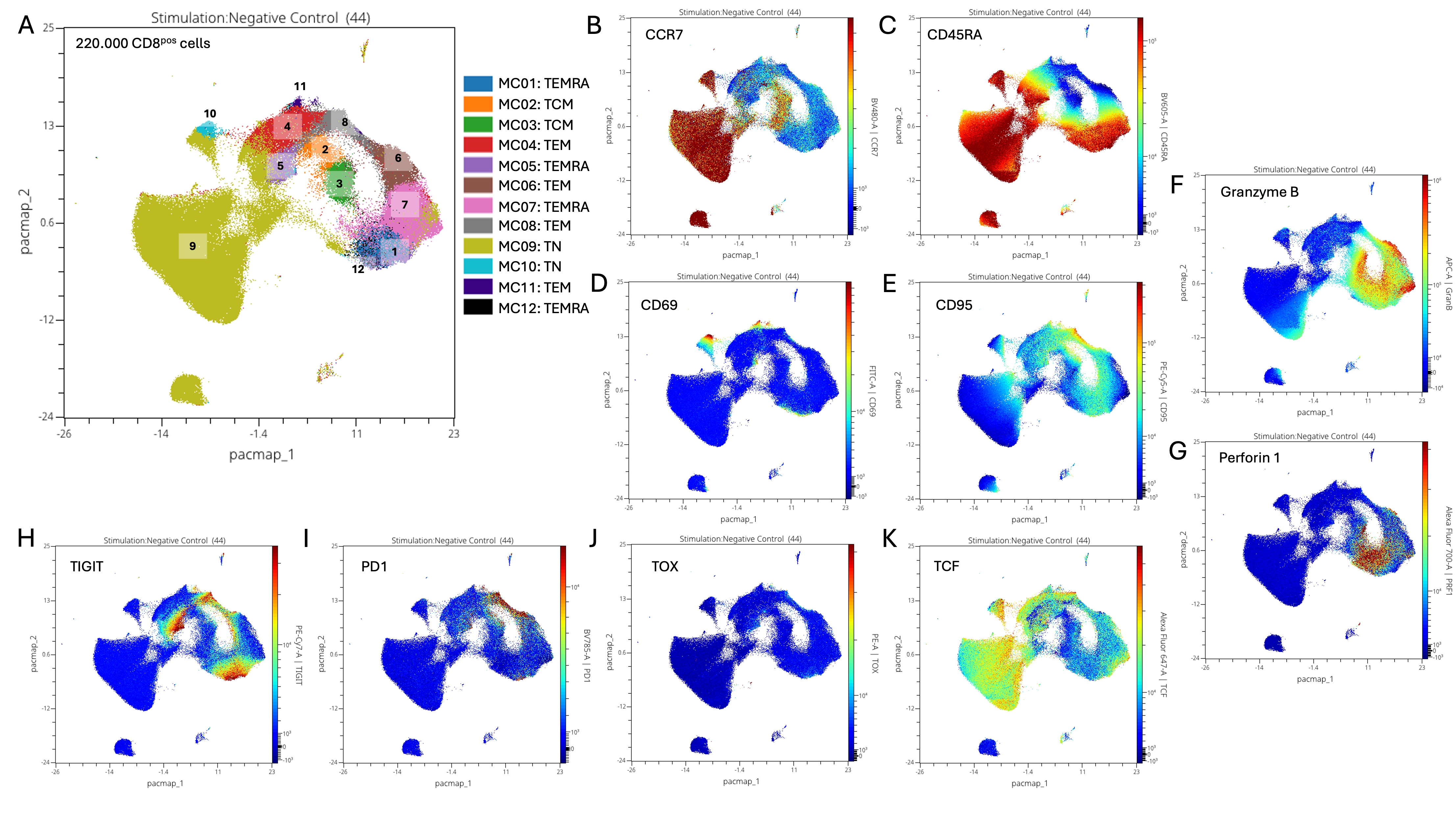

Supplement: Supplement 13 [file media-13.jpg]

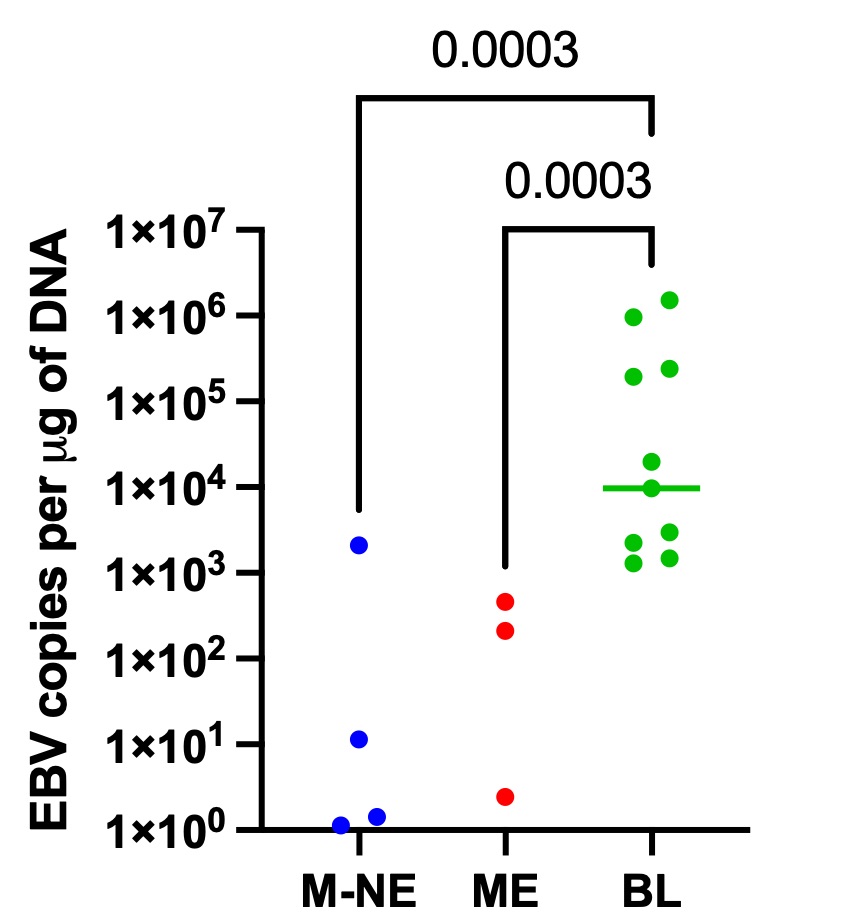

Supplement: Supplement 14 [file media-14.jpg]

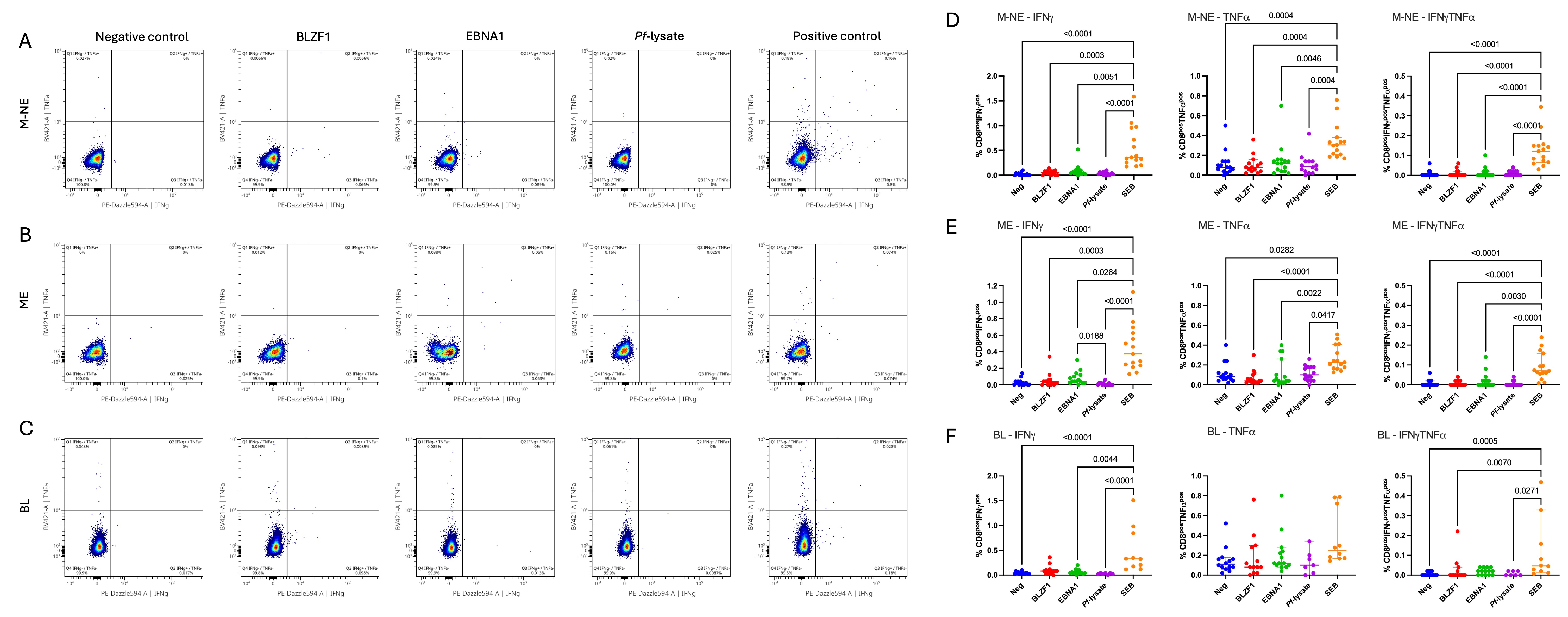

Supplement: Supplement 15 [file media-15.jpg]

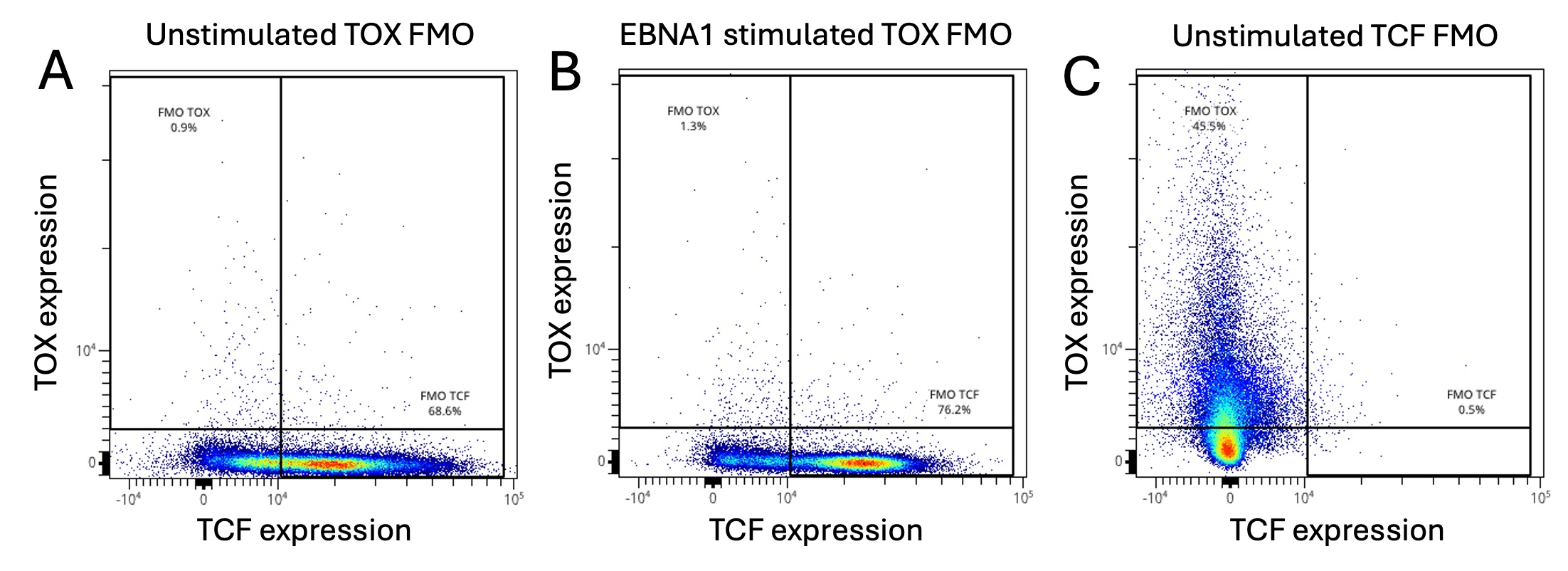

Supplement: Supplement 16 [file media-16.jpg]

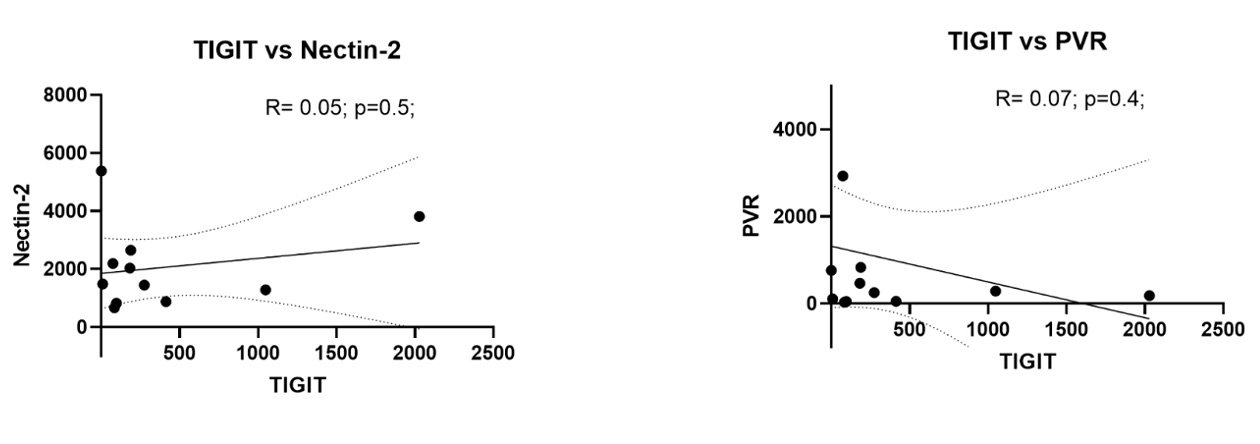

Supplement: Supplement 17 [file media-17.jpg]
